# Issues in federated learning: some experiments and preliminary results

**DOI:** 10.1038/s41598-024-81732-0

**Published:** 2024-12-02

**Authors:** Jamsher Bhanbhro, Simona Nisticò, Luigi Palopoli

**Affiliations:** DIMES, University of Calabria, 87036 Rende, Italy

**Keywords:** Federated learning, Data heterogeneity, Client weighting, Model personalization, Data privacy, Engineering, Mathematics and computing

## Abstract

The growing need for data privacy and security in machine learning has led to exploring novel approaches like federated learning (FL) that allow collaborative training on distributed datasets, offering a decentralized alternative to traditional data collection methods. A prime benefit of FL is its emphasis on privacy, enabling data to stay on local devices by moving models instead of data. Despite its pioneering nature, FL faces issues such as diversity in data types, model complexity, privacy concerns, and the need for efficient resource distribution. This paper illustrates an empirical analysis of these challenges within specially designed scenarios, each aimed at studying a specific problem. In particular, differently from existing literature, we isolate the issues that can arise in an FL framework to observe their nature without the interference of external factors.

## Introduction

In the rapidly evolving field of machine learning (ML) and artificial intelligence (AI), the methodologies for developing and deploying models have undergone a significant transformation. Streamlined frameworks have simplified the training process, making basic and intricate ML tasks more accessible and allowing for their execution with remarkable ease and effectiveness. However, a critical challenge lies at the heart of these advancements: data acquisition and management. Traditionally, ML models have relied on centralized data repositories, requiring data to be moved to the model - a practice fraught with privacy, security, and accessibility issues. This centralized approach has in fact received increasing scrutiny due to its potential for breaches and vulnerabilities. For instance, Alaqra et al.^1^ highlight the risks associated with centralized data management in sensitive medical data, while Brauneck et al.^2^ emphasize the need for privacy-enhancing technologies like FL to address these challenges.

Indeed, FL emerges as an innovative, decentralized approach to ML, fundamentally transforming traditional data-centric model training methodologies. This approach redefines the process by bringing the computational model directly to the source of data generation, such as mobile devices or edge servers, thereby negating the need for central data aggregation^3^. Such a strategic pivot not only significantly bolsters data privacy and security but also facilitates instantaneous updates and enhancements to models by harnessing the diverse array of data naturally generated in user environments, without transferring sensitive information away from its origin. Thus, FL marks a substantial advancement in ML and AI, promoting a collaborative, inclusive framework for model development. It allows for an extensive network of devices to partake in model training, each contributing with updated parameters like weights or gradients instead of exposing raw data, ensuring that personal and sensitive data remains securely within the device, aligning with rigorous data protection standards.

However, the benefits of FL extend beyond enhancing privacy and security. Indeed, FL optimizes bandwidth usage by transmitting minimal model updates rather than extensive volumes of raw data, making it an efficient solution even in bandwidth-constrained environments^4^. Furthermore, by leveraging data from a broad spectrum of sources, FL ensures the development of models that are diverse, robust, and representative of real-world scenarios, thereby improving the accuracy and reliability of ML applications. This decentralized model also showcases superior scalability and efficiency compared to traditional training methods, utilizing computational resources across a vast network of devices and reducing dependency on centralized servers. This is particularly advantageous in remote or resource-constrained settings, where access to central servers may be challenging, thus democratizing data and computational resources across various applications.

Despite its considerable advantages, FL faces several challenges, including data heterogeneity, data privacy, resource constraints, and model complexity. Data heterogeneity, where data across devices vary significantly, can adversely affect model performance and convergence, necessitating suitable aggregation techniques^5^. Privacy concerns, while mitigated by FL’s decentralized nature, still require robust encryption and secure multi-party computation methods to prevent information leakage during the learning process^6^. Resource limitations on participant devices pose another challenge, as FL must balance computational and communication costs without compromising model training or accuracy^7^. Lastly, the complexity of FL models, which must be designed to operate efficiently across diverse and potentially resource-constrained environments, calls for innovation in model architecture and compression^8^. Addressing these issues is crucial for the advancement and widespread adoption of FL technologies.

In this paper, we aim to practically analyse the multifaceted challenges of FL by constructing various FL environments to study the conditions in which they arise. This approach allows us to examine each issue separately, facilitating the controlled analysis of their impacts on the system’s overall performance and reliability. Through the application of targeted solution techniques within these distinct environments, we intend to shed some light on the prevalent issues of data heterogeneity, data privacy, resource constraints, and model complexity that are fundamental to FL. Furthermore, our study will document the exploration of these challenges and will bring out the strengths and weaknesses of some solutions proposed to address them. This will hopefully provide a clearer idea of FL’s open problems.

By conducting an analysis and empirical evaluation of these solutions in various scenarios, our goal is to underscore the viability and impact of approaches proposed to address the considered issues. This will provide researchers and practitioners with an analysis of the open problems in FL that need to be addressed and ideas about possible ways to face them.

To summarize, the main contributions of this paper are the following.

- We have singled out the problems found in the FL approach by the current literature and practically experimented on them considering also some of the solutions proposed to mitigate them.
- We have directly dealt with the FL framework as it behaves the same across all types of application domains. Therefore, our analysis is of the general kind, not sticking around a specific domain or technology. Rather, it focuses on using different datasets to evaluate general FL.

All in all, this empirical analysis aims to group all the issues observed in the FL field to provide an overview of its strengths and weaknesses. The rest of the work is organized as follows: “Literature review” Section reports on existing literature, “A study of FL issues” Section describes FL issues and some solutions proposed to try to solve them, “Discussion” Section comments on the analyzed features and, finally, “Conclusion” Section concludes this work.

## Literature review

The concept of FL has significantly gained traction since its introduction by McMahan et al. in their pioneering 2017 paper^9^, “Communication-Efficient Learning of Deep Networks from Decentralized Data.” This foundational research highlighted FL as a pivotal response to the escalating concerns over data privacy and security inherent in traditional ML frameworks. Traditional ML paradigms often rely on centralized data aggregation, which amplifies the risk of data breaches and misuse. FL emerges as a compelling alternative by facilitating model training across dispersed datasets, thereby inherently bolstering the privacy and security of data.

The critical need for FL becomes even more apparent when considering the ramifications of high-profile data breaches. A notable example provided by Sweeney^10^ detailed an incident involving the Massachusetts Group Insurance Commission, where “anonymized” health records were re-identified, exposing significant privacy breaches. This incident highlights the inherent vulnerabilities of centralized data storage systems used in ML, emphasizing the potential of FL to mitigate such risks. Indeed, in FL it is not required to share the data records since local models share with the global model only the updated weights to aggregate, from which it is harder to reverse-engineer sensible information. Moreover, in FL frameworks each client owns only a small portion of data making it harder to attack data stored in multiple local storage, often with diverse formats. By leveraging decentralized data processing, FL presents a viable path to crafting ML models that are not only efficient but also prioritize privacy, addressing the growing demand for technologies capable of protecting sensitive data against sophisticated cyber threats.

While FL inherently enhances data privacy, the risk of information leakage through model updates remains a concern. Techniques such as differential privacy, as discussed by^11^, offer solutions to further secure FL against potential data privacy breaches, ensuring that the model learning process does not compromise sensitive information.

One of the inherent challenges in FL is data heterogeneity. The data distributed across devices often vary significantly, making it challenging to train a model that performs well across all nodes. Li et al.^5^ discussed strategies to manage this heterogeneity, emphasizing the need for algorithms that can handle diverse data distributions effectively.

The complexity of models in FL can also pose significant challenges, particularly in terms of computational resources and efficiency. Smith et al.^8^ explored methods to reduce model complexity without compromising the performance, highlighting the importance of optimizing models for the unique constraints of FL environments.

Also, effective resource management is crucial in FL, given the diverse and potentially resource-constrained devices involved in the learning process. Konečný et al.^12^ addressed this challenge by proposing strategies for efficient communication and computation, aiming to minimize the resource demands of FL on participating devices.

Through properly addressing these challenges, FL continues to evolve as a robust framework for ML, promising significant advancements in privacy, security, and efficiency. The ongoing research and development in this field, as highlighted above, are crucial for realizing the full potential of FL, making it a cornerstone technology in the era of data-driven decision-making. Following the discussion on the importance of effective resource management in FL, it is noteworthy that several other studies further explored various challenges within FL, offering innovative solutions and insights. These studies are summarized in Table 1, which provides a concise overview of recent research focusing on issues such as data privacy, efficiency, and the application of advanced techniques like blockchain and differential privacy in FL.

From the content of the previously referred table, it is possible to observe how some works either focus on solving specific issues of FL^13–19^, survey the literature on one or more aspects^20,21^ of FL, theoretically discuss possible issues^22^, or discuss FL tailoring it to specific applications^23^. Differently from all of that, our contribution looks at FL’s framework without referring to a specific context, in order to study the issues linked to its general structure. Moreover, our goal is also to show when (and when not) these issues arise, through experiments properly designed and held in simple controlled environments.

In the forthcoming sections of this paper, we will embark on a practical analysis of the challenges identified within the FL framework by the recent literature. This analysis aims to not only delve deeper into the intricacies of these challenges but also to conduct some solution evaluation through empirical study. By leveraging some real-world datasets and scenarios, we intend to scrutinize the effectiveness of existing strategies to enhance the efficiency, scalability, and security of FL systems. This practical exploration will contribute to the ongoing efforts to optimize Federated Learning for several application contexts.

**Table 1:** Summary of some studies on federated learning issues.

References	Issue addressed/described	Techniques used	Key findings
^13^	Data privacy	Blockchain, federated learning	Demonstrated improved performance and efficiency in data sharing
^14^	Data privacy	Privacy-preserving data aggregation	Proved efficiency in computing and communication resources while protecting users’ privacy
^15^	Data privacy	Blockchain, differential privacy, homomorphic encryption	Designed multiple data protection methods in IIoT settings
^22^	General FL challenges	This describes survey of FL issues	Discussed recent advances and presented a collection of open problems
^21^	Data privacy	Data partitioning	Provided a comprehensive review and categorization of FL systems
^16^	Data privacy	Partial model personalization	Described the importance of FL in FinTech for confidentiality
^17^	Data heterogeneity, data privacy	Differential Privacy	Explored privacy protection in FL with non-IID data and designed a novel algorithm
^18^	Weighted aggregation challenges in federated learning	The authors introduce a concept called client coherence and propose a new method named FedLAW (Federated Learning with Learnable Aggregation Weights).	Improved model generalization with dynamic weight adjustments. Discovery of a global weight shrinking effect, similar to weight decay, which can enhance generalization by having the sum of weights be less than 1
^19^	Data heterogeneity	To improve accuracy in federated learning, the authors suggest sharing a small set of data across all devices	This paper mentions that sharing 5% global data impacts accuracy by a good amount
^20^	Data poisoning and privacy breaches	Anomaly detection and secure aggregation	A survey on federated learning security that highlighted potential vulnerabilities in communication channels, data manipulation, and the central server. The paper also identified various attack methods and emphasized the need for further research to ensure robust security for a wider adoption of this technology
^23^	Challenges in the context of FL and edge computing	The article discusses the FL and edge computing to develop ubiquitous intelligence in the 6G network	Highlighted the potential of combining FL and edge computing to enhance UI in 6G networks

## A study of FL issues

Before diving into details about analyzing FL challenges, it is crucial to understand the foundational elements required for a successful FL setup. A robust FL framework demands significant computational power in both clients and global server, secure and reliable communication for model updates, and a carefully considered global model architecture to integrate diverse client models effectively. Ensuring data consistency across clients further enhances the model’s ability to learn universally applicable patterns, thereby boosting overall system performance. Furthermore, addressing data privacy proactively, through techniques like differential privacy, fortifies the FL system against potential breaches.

In this section, we implement and analyze specific issues related to FL. For each FL issue, we present a FL environment designed to analyze the issue. Additionally, we detail the techniques or solutions that we found to be effective in addressing the issue arising in our controlled environments. Our goal is to study each issue individually to provide some useful insights into them. Therefore, we created specific simple environments tailored to each problem. Below, we will discuss each issue along with the corresponding solutions.

Since in all our experiments we deal with classification, we use accuracy as a quality metric, which is widely used also in the evaluation of general machine learning classification algorithms. Considering a set of *n* samples, the accuracy is computed as:

1 $$\begin{aligned} \text {Accuracy} = \frac{TP}{n} \end{aligned}$$

where $TP\le n$ is the number of correctly classified samples.

In FL, accuracy is typically measured in two ways: local accuracy and global accuracy. Local accuracy refers to the accuracy of each client, measured on its own local test dataset. In contrast, global accuracy is determined by evaluating the global model using the test data from all clients. This dual approach allows for a comprehensive assessment of the model’s performance, both individually for each client and collectively across the entire system. In our experiments, when we refer to global accuracy, we mean to use a test data sample extracted before splitting the data among the clients.

In cases where we use the same data for each client but in different forms (IID and non-IID), we use the same testing datasets for each client to evaluate performance. In cases where we use different data, such as assigning a few digits images from the MNIST database^24^ to one client and different digits images to other clients, we divide the data into subsets and use 20% of the data as testing data for each specific local client.

### Data heterogeneity issue

**Fig. 1:**
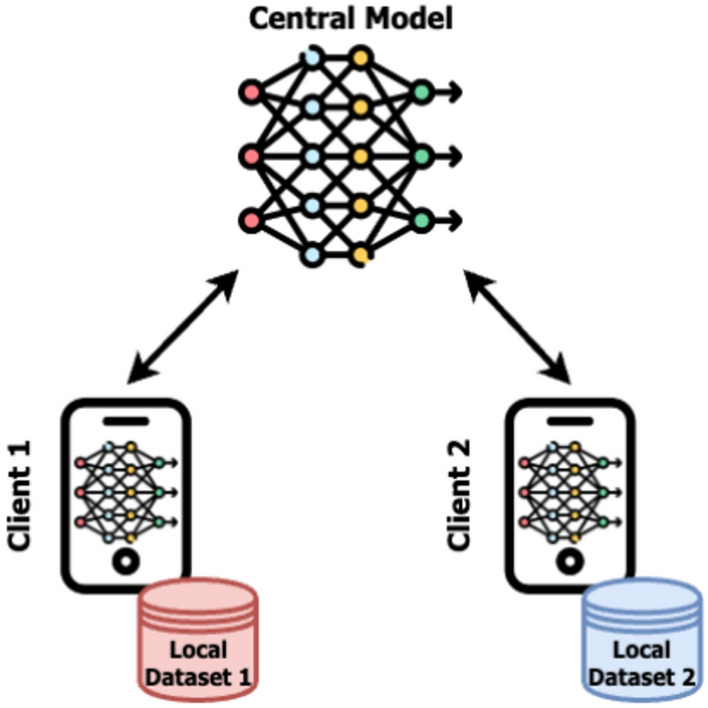
FL environment for analyzing data heterogeneity issue. In the considered context, two clients communicate with a central server that maintains a global model. In the first scenario, this environment was trained using IID data, while the second scenario used non-IID data.

In FL, data across clients may be non-IID (not independently and identically distributed), unbalanced, and highly variable in terms of both quantity and quality. Such variability can result in models that perform well on some clients but poorly on others. To examine the issue of data heterogeneity, we simulated a federated learning environment to train a convolutional neural network (CNN) model using PyTorch. Figure 1 depicts the structure of the FL environment considered in the experiment we are going to discuss in this section.

In this setup, we implemented a simple federated framework involving two clients, trained using MNIST dataset data from LEAF^25^, adapting it for FL tasks. Each client’s model, as well as the global model, follows a uniform structure consisting of straightforward CNN layers. This ensures consistency in model architectures across the federated network, allowing us to focus only on the effects of data distribution rather than on architectural differences. After developing and training the models, we aggregated the outcomes to obtain global model parameters. PyTorch’s *torchvision.datasets* library was used to load the dataset and *transforms.Compose* was used for data preprocessing, including conversion to tensors and normalization. To have a clear idea about the results achieved with aggregation performed employing simple averaging, as well as about their stability, we trained and tested models 10 times in order to compute the mean accuracy value together with its standard deviation.

**Fig. 2:**
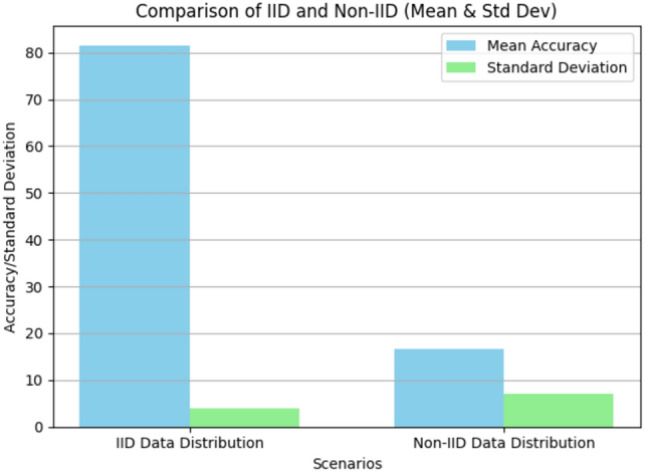
Comparison between the accuracy of the same FL framework trained with data having an IID distribution and data having a non-IID distribution.

This test resulted in a mean accuracy of $82\%$ with a standard deviation of 3.08 in the scenario without data heterogeneity (IID data distribution). Then, to observe what happens in the non-IID scenario, we considered the same FL environment (the one of Fig. 1) of the previous test together with the same data albeit in different arrangements. Indeed, while for the IID scenario, the clients have a balanced half-split of the data, for the non-IID scenario, we created a more imbalanced data distribution across clients. We achieved this by iterating through the dataset’s labels and creating separate subsets using list comprehensions. Client 1 received data points with labels 0-4 (images of digits 0 to 4), while Client 2 received labels 5-9 (referring to digits 5 to 9). Subset objects were used again to create data loaders (DataLoader) for each client, ensuring shuffled batches during training. From the 10 runs we performed, it has been observed how the mean accuracy always dramatically drops, achieving a mean accuracy of $17\%$ with a standard deviation of 7.06. It is useful to keep in mind that we used limited epochs, specifically 3, in order to observe the issue in boosted form. Indeed, to keep things controlled we are dealing with a very small environment and, hence, for better highlighting the difference between the two settings. In particular, we used limited epochs for otherwise, with many epochs running, accuracy would get anyway close to $100\%$ due to the simplicity of the federated data we used, thus hiding the differences we are looking for. In IID scenarios, if all clients have IID data, they are supposed to have similar weights, making it easier for the global model to achieve the highest accuracy. Figure 2 reports the previously discussed results.

Once stated and experimented with the problem of data heterogeneity, in the following, we discuss two solutions referring to the number of clients and client weighting. Additionally, we will discuss model personalization, which considers data diversity from a different perspective.

#### Number of clients

If there are fewer clients, as described in the scenario introduced in “Data heterogeneity issue” Section, and a significant amount of non-IID data, this, as shown earlier, will likely significantly decrease the performance of the FL environment. We made a minor adjustment to the FL environment (Fig. 1) by adding two more clients and, then, we trained the system with non-IID data using the same settings as described above. By this change in the number of clients, we preliminary observed some variations in accuracy. We then modified the environment by adding varied clients and tested the impact of the number of clients on performances in non-IID scenarios. We experimented on this aspect using MNIST data, which is a simple dataset, and CIFAR-10 (Canadian Institute For Advanced Research-10)^26^, which is a bit more complex. To more properly analyse the impact of the number of clients on the environment where the data is non-IID or has high heterogeneity, we performed experiments on several FL environments. We created FL environments consisting of 2 clients, 4 clients, and 6 clients. For the non-IID scenario and to analyze the idea that an increase in the number of clients will improve accuracy, we practically tested all the FL environments on both the MNIST and CIFAR-10 datasets. For the non-IID scenario, we created non-IID data subsets. Importantly, these subsets were identical across all FL environments. In simpler terms, to create the subsets, we used the same set of data points and maintained their non-IID characteristics in the sampling. In particular, for the two-client scenario, each client had a different data distribution. Recalling that both the considered datasets are composed of 10 classes, in the four-client and six-client scenarios we created different data distributions for each client by equally distributing the samples for 2 and 4 classes respectively, while the remaining classes are unevenly distributed among the clients. More in detail, in the four-client setting, the majority of samples belonging to classes 0 and 2 are assigned to client 1, the majority of the samples belonging to classes 3 and 5 to client 2, the majority of samples labelled as 4 and 7 to client 3, finally, a larger amount of samples from classes 8 and 6 are assigned to client 4. Instead,in the six-clients scenario, a larger amount of samples of class 0 are assigned to client 1 and, similarly, for class 2 to client 2, for class 3 to client 3, for class 4 to client 4, for class 5 to client 5, and finally, the majority of samples of class 6 are associated with client 6.

We ran each experiment 10 times to check the stability of our results. Hence, the table shows the average accuracy of 10 runs of each environment followed by their standard deviation. Table 2 shows the differences in the non-IID performance with different numbers of clients for the two considered datasets. A glimpse at the results reported in the table shows how, even when dealing with easy-to-handle datasets such as MNIST and CIFAR-10, having so heavily different data distributions across different local clients, as that simulated in the previous experiment, heavily affects the performances of the global model. The reason for the observed behavior stems from the combination of weights codifying diverse information.

**Table 2:** Central model accuracy comparison with respect to the number of clients in a non-IID scenario

Total clients	Epochs	Accuracy on MNIST non-IID	Accuracy on CIFAR-10 non-IID
2	5	89.9 ± 1.2	29.59 ± 2.2
4	5	63.35 ± 1.73	21.93 ± 1.45
6	5	50.05 ± 2.52	10.79 ± 0.72

Table values report the means and the standard deviations.

The simple nature of the datasets we used up to this point does not allow us to increase the number of clients ensuring data is in non-i.i.d forms. Thus, in order to perform the same experiment by considering a more complex and realistic scenario involving a greater number of clients, we used the MRI dataset^27^, which is a medical imaging dataset containing images from brain magnetic resonances from healthy patients and patients affected by brain tumors. This one undoubtedly represents a more challenging dataset from a data heterogeneity point of view. We note here that this dataset is particularly interesting for the class it refers to, as the application type they consider. Indeed, FL’s decentralized approach appears particularly suitable for medical applications^28^.

In more detail, this dataset collects 7023 human brain magnetic resonance images from people not affected by tumours and people affected either by glioma, meningioma or pituitary gland tumors. This dataset comes as a combination of three different data sources, so it is of specific interest with respect to the data heterogeneity issue we are discussing.

To build up our experiment, the data has been divided across several local clients varying from 5 to 30, shaping the data subsets in such a way as to mimic real-world non-i.i.d. conditions. So, we divided data in a way that makes the clients receive a larger volume of data from certain classes than from others, which results in an imbalanced and non-uniform data distribution. This reflects real-world scenarios where the data available to different clients may be biased or skewed toward particular labels or features. The peculiarity of this kind of dataset is that the heterogeneity comes not only from the observable classes (which are less in number than the previously considered simple datasets) but, more importantly, from the variety each class has in itself, due to the high data complexity.

The results reported in Table 3, providing the average accuracy of the global model over 10 runs, show that, as the number of clients increases, the global model’s accuracy has an increasing trend reaching its maximum accuracy in correspondence of the maximum performance ($66.88\%)$ of clients considered in the experiment. These results give evidence of the benefits of a larger and more diverse data distribution, which seems to conflict with the results observed on the MNIST and CIFAR-10 datasets. The take-off message of these, apparently, opposite results is that heavily heterogeneous data distributions, like that observed in the experiments with MNIST and CIFAR-10 involving 4 and 6 clients, led to drops in accuracy even when the number of clients increases. Indeed, in such a challenging setting, the global model’s performances are affected by the difficulty of combining so much variegated information. This emphasizes a trade-off: while increasing the number of clients enhances data diversity and improves the model’s generalization, excessive heterogeneity can hinder convergence and reduce overall accuracy, challenging the model’s ability to effectively generalize.

**Table 3:** Average test accuracy across different numbers of clients using MRI dataset.

Number of clients	Average test accuracy (%)
5	49.43
10	60.95
15	63.72
20	63.54
30	66.88

#### Client weighting

There are several techniques available for addressing non-IID data challenges in federated learning, such as the local learning technique called FedAlign^29^, data augmentation and data privacy processing described in reference^30^, and client clustering^31^. Among the most utilized methods in FL is client weighting (see reference for further insights^32^), where updates from clients are not treated equally but are instead calculated based on specific criteria. These criteria can include the volume of data a client contributes, the quality and representation of their data, the performance of a client’s update when evaluated on a validation set, or considerations of fairness to ensure equitable representation of all clients in the aggregated model.

In our experiments, whose results are reported in Table 4, to explore the impact of client weighting on accuracy in FL environments, we consistently applied a simple client weighting strategy in the framework of Fig. 1 consisting of two clients. In particular, in the here adopted weighting strategy, each client’s weight corresponds to its data contribution (we will describe it more thoroughly in “Model aggregation: simple versus weighted averaging” Section). Initially, we assigned a data split dominated by IID data (80% IID to Client A and 20% non-IID to Client B). As expected with this data distribution (row 1 in the table), the accuracy was very high because Client A’s IID data significantly improved the global model’s performance. As we progressed through the experiments (rows 2–4 in Table 4), the non-IID data presence in the data distribution became more relevant. This, along with the application of client weighting, resulted in a significant decline in accuracy. For example, with a more unbalanced distribution of $60\%$ non-IID and $40\%$ IID (row 4), the mean accuracy reaches a value of $23.1\%$ for CIFAR-10 and $53.58\%$ for MNIST.

We would like to mention that both MNIST and CIFAR-10 have relatively few classes. Also, we are keeping the same format of non-IID across each run and each setting. We have observed that when there is significant dominance of the non-IID weights, the performance of the model improves after a few epochs due to the less diverse nature of the weights to aggregate. Indeed, the IID data have a small impact on the weights update operations. However, the dominance of non-IID data impacts other clients in the FL environment negatively. An evidence of this claim is provided by the accuracies of Client A for MNIST and CIFAR-10 reported in the last two rows of Table 5, where the performances of the local clients after the client weighting application are presented (we recall that Client A represents the client with IID data, while Client B represents the client with non-IID data). The first row of the table shows that Client B, with non-IID data, has an accuracy of 28.99% for CIFAR-10, and 86.9% for MNIST. The table clearly shows how client weighting influences the accuracies for local clients, highlighting the negative impact of client weighting in contexts lacking fairness (in terms of uniformity of data available to each client).

**Table 4:** Impact of client weighting based on data volume distribution on global accuracy of FL Environment.

IID weight %	Non-IID weight %	MNIST accuracy (%)	CIFAR-10 accuracy (%)
80	20	94.08 ± 1.36	41.2 ± 1.9
70	30	82.42 ± 7.41	30.8 ± 4.6
60	40	50.94 ± 13.49	23.83 ± 3.9
40	60	53.58 ± 12.38	23.1 ± 4.7
30	70	75.29 ± 11.27	34.0 ± 2.11
20	80	93.35 ± 1.57	42.6 ± 1.7

Table values report the means and the standard deviations.

**Table 5:** Accuracies of Client A and B for MNIST and CIFAR-10 across different IID/non-IID settings.

IID %	Non-IID %	Client A (CIFAR-10) %	Client B (CIFAR-10) %	Client A (MNIST) %	Client B (MNIST) %
80	20	45.57 ± 1.92	28.99 ± 2.01	96.32 ± 0.27	86.47 ± 1.59
70	30	44.15 ± 1.75	32.50 ± 1.90	95.435 ± 0.59	89.648 ± 2.14
60	40	41.92 ± 2.58	36.70 ± 2.24	95.292 ± 0.49	93.782 ± 0.62
40	60	37.80 ± 2.57	41.69 ± 2.44	93.263 ± 0.65	95.199 ± 0.61
30	70	32.57 ± 2.22	44.13 ± 2.52	89.826 ± 1.70	95.444 ± 1.81
20	80	28.30 ± 3.61	46.56 ± 2.13	86.485 ± 1.99	96.262 ± 0.33

Table values report the means and the standard deviations.

#### Model personalization

Another interesting opportunity in FL is model personalization. This strategy involves training a central model on all the available data. Then, this central model is refined, to create models specific to each client.

In more detail, personalization works by fine-tuning a global model on individual clients’ local data, allowing it to adapt to the specific data distribution of each client. Thus, in a FL framework, the global model is preliminary trained with the updates provided by at least a portion of the local models and, then, the clients fine-tune a copy of the so-trained global model using only clients’ local data. This customization allows the model to better capture the unique data characteristics and patterns of each client, often leading to significant improvements in both global and local accuracies. However, it is important to remember that this approach requires more computing power on the clients’ devices and more communication to exchange reasonably detailed model updates. The reader can find more details in Reference^16^.

In our experiment, which considers the CIFAR-10 dataset, we also studied model personalization. We began by copying a trained global model, ensuring both Client A and Client B started with a basis learned from the entire dataset. Next, we fine-tuned these copies on each client’s local data. For Client A, this involved fine-tuning the model on its $80\%$ share of IID data, while for Client B, the data used for fine-tuning is the $20\%$ share of non-IID data. This additional training, performed over 5 epochs, is targeted to adjust model weights to better align with the unique characteristics and patterns present in each client’s data. By incorporating these personalized models, we aimed to capture insights gleaned from each client’s specific data. After fine-tuning, we measured the accuracy of each personalized model on a test data set reflecting the peculiar data distribution of each client. This ensures that the evaluation reflects the performance computed on each client’s unique dataset. Satisfyingly, this method of model personalization, applied within our existing FL setup, led to an accuracy boost. Client A’s personalized model achieved an accuracy of $49.15\%$ when the data was non-IID, and Client B’s model reached $49.47\%$ when the data was IID. The results of the Table 6 show how, after personalization, the framework performs better compared to Table 5 (which reports client’s performances without model personalization) on local clients’ data, witnessing an improvement in the generalization on client-tailored data. This finding witnesses the capabilities of model personalization in FL, suggesting its ability to substantially enhance model performance by leveraging the richness and variety of distributed data. More specifically, model personalization greatly helps clients with less data by providing them with a better initialization for their weights.

**Table 6:** Impact of data distribution and personalization on local performances of CIFAR-10.

Configuration (IID, Non-IID)	Client A with personalization	Client B with personalization	Global accuracies
80% , 20%	58.80 ± 0.55	49.47 ± 3.18	46.75 ± 1.32
70% , 30%	56.99 ± 0.96	51.89 ± 1.36	45.84 ± 3.02
40% , 60%	53.80 ± 1.60	56.38 ± 1.42	47.05 ± 1.60
30% , 70%	54.20 ± 2.70	59.89 ± 2.58	52.84 ± 3.02
20% , 80%	49.15 ± 0.98	58.80 ± 0.76	48.09 ± 1.32

Table values report the means and the standard deviations.

### Model aggregation: simple versus weighted averaging

In the previous section, by training a FL framework in properly designed settings we have analyzed the impact of data distribution and data-dimension-based client weighting strategy on the accuracy reached by the central model. From this analysis, involving the training of the same model on data sampled from the same dataset, it emerged how data distribution affects the quality of the FL framework. Moreover, the results of the experiment concerning the above-described weighting strategy suggest how important it is to design a weighting strategy giving more importance to more reliable and informative updates. Thus, particular attention must be given to selecting aggregation methods in FL environments to enhance model performance and accuracy.

The simplest averaging technique for model aggregation is represented by a simple arithmetic average giving the same importance to each model update. Its weak point is the equal contribution of all the clients, whose updates are not always in a similar fashion and have the same quality level.

**Fig. 3:**
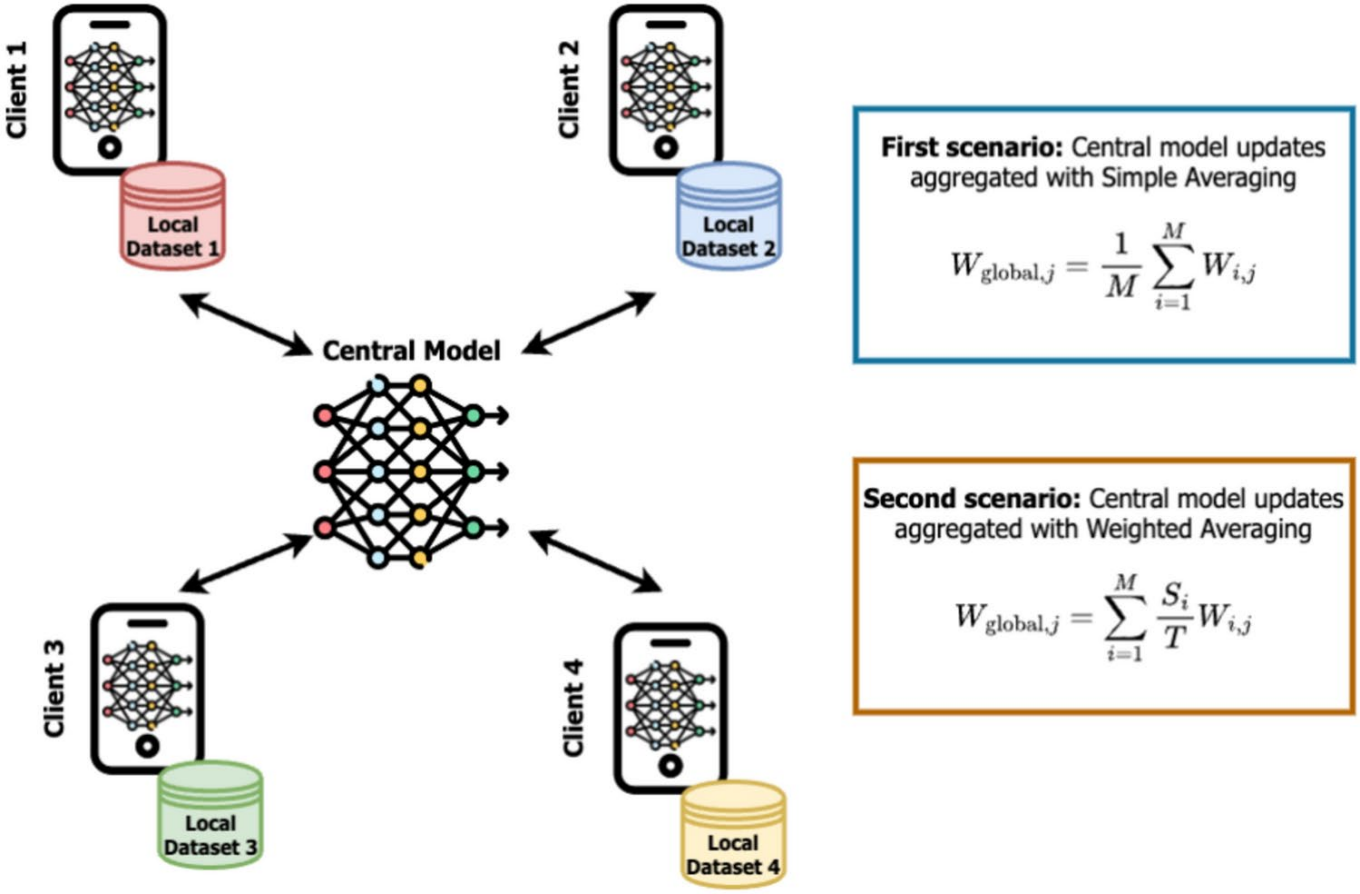
On the left of the figure, a FL framework consisting of four clients with their own dataset. On the right, the two scenarios considered for client weighting.

The first step to improve the weighting strategy based on simple averaging, whose results are shown by Table 4 relative to the last experiment of “Data Heterogeneity issue” Section, is to weight each client according to the size of the data used for its training step.

2 $$\begin{aligned} W_{\text {global},j} = \sum _{i=1}^{M} \left( \frac{S_i}{T} \right) W_{ij} \end{aligned}$$

**Fig. 4:**
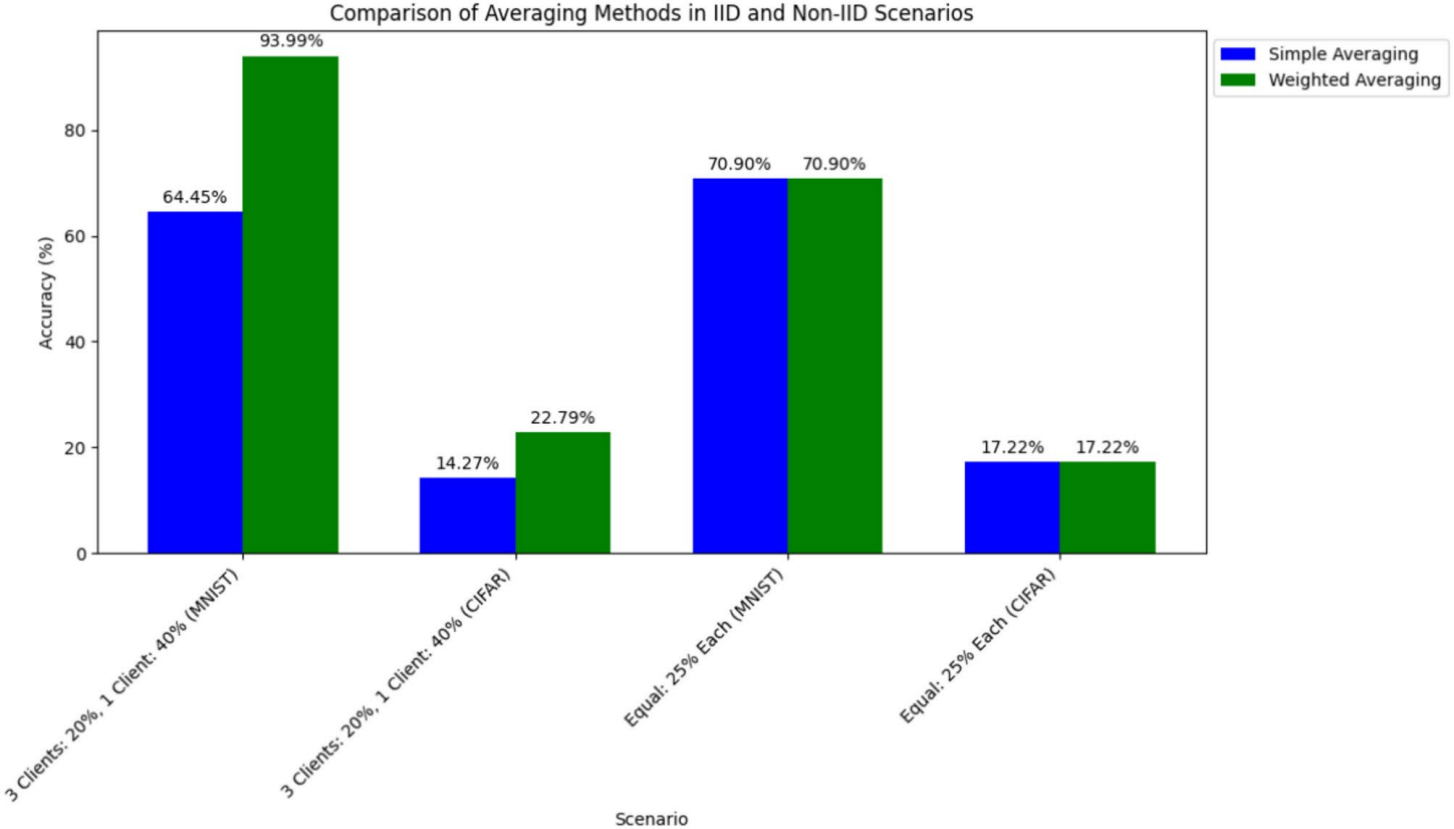
Comparison of the accuracy achieved by FL central model obtained according to different weighting strategies.

Equation 2 reports the update for the *j*th weight in the global model $W_{\text {global},j}$. It considers the contribution from *M* clients, where each client *i* has a different training data set containing $S_i$ samples. The value $T=\sum _{i=0}^{M}S_i$ represents the total number of samples the FL environment has across all the clients. With this formula, the contribution of the *j*th weight $W_{i,j}$ of the client *i* to the *j*th weight of the global model is scaled by the ratio between the size of its dataset $S_i$ and the total amount of data *T* (whose value ranges for 0–1). This weighting technique ensures that clients with larger datasets have a greater influence on the final model, following the logic that clients having more data volume can improve training effectiveness. The reverse of the coin is that it considers only quantity discarding the quality of the data. Thus, when a client has a huge amount of data but this data has poor quality, it lowers the efficacy of the update (with respect to simple averaging) because such a client has a larger influence on the global model weights. This phenomenon can be observed in the first 3 rows of Table 4, from which it is clear how a poor data quality (given by its non-IID distribution) hugely affected the accuracy of the global model.

Then, we trained the environment with four clients using data in different scenarios. We used data from the MNIST and CIFAR-10 datasets to evaluate the performance of a FL framework and both averaging methods. Initially, we trained the FL environment in a scenario where we gave three clients 20% of the data each in normal form, and we gave 40% of the data to the fourth client, evaluating both the framework and averaging methods. Subsequently, we retrained the same FL environment using an equal percentage of data, like 25% to each, and again evaluated the framework and averaging methods. Our environment is shown in Fig. 3. From the experiments, we found that when the data portion divided is similar, both methods work in the same way, and when one client has more data, simple averaging performs less effectively than weighted averaging. Figure 4 reports the comparison of results between the two methods for the two datasets, which shows how, in all the considered environments, weighted averaging performs better compared to simple averaging. Obviously, in a situation in which all the clients have the same amount of data, the accuracy achieved by the weighted aggregation is the same as that achieved by simple aggregation (see the second half of the plot in Fig. 4) because the weights are the same for each client.

### Regarding data privacy

FL has an advantage because of the decentralized approach and also provides security on the data, as data is not shared individually, regardless of domain. And, indeed, one of the paramount advantages of FL is its privacy-preserving mechanism. In FL, privacy is inherently protected as long as there are no vulnerabilities in the communication process. However, while FL is considered secure from a data perspective, if an attacker manages to compromise the communication channels or gains iterative access to the updates shared by the models in a FL environment, they could potentially analyze the patterns of the data points by examining the continuous gradients. This vulnerability is due to weights exchange that is part of FL frameworks thus it potentially affects each FL system, beyond the peculiarities each specific context has. In this section, we consider the data privacy issue in a generic FL framework, to keep things simple and not be influenced by the distinctive characteristics of specific application contexts.

To illustrate, several methods are available to mitigate those risks, including differential privacy (both local and global) and encryption techniques like secure aggregation. These methods enhance privacy by adding encryption at the client level and decryption at the global model level. Additionally, training client models with noisy data can prevent the sharing of actual parameters with potential attackers. In the following we analyze differential privacy methods by first observing how gradients computed on randomly generated synthetic data points changes after the application of these methods, and then studying how they changes the performance achieved on the MNIST dataset.

#### Differential privacy

The Differential Privacy (DP) framework is designed to provide strong privacy guarantees for individuals in a dataset. It works by adding a certain amount of noise to the data or the clients’ outputs, ensuring that the removal or addition of a single database item does not significantly affect the outcome. This makes it difficult for attackers to infer information about any individual data point. The reference^33^ has described the mathematical background of DP. This method can be applied to the outcomes of the FL clients and/or of the global model. In the first case, we are dealing with *Local Differential Privacy (LDP)*, where noise is added to the client’s updates before they are sent to the server. This approach ensures that each user’s data is privatized at the source, offering strong privacy guarantees. Differently, with *Global Differential Privacy (GDP)*, the noise is added at the level of a global model so to the updates that are going to be transmitted to the local clients.

To analyze the effect of LDP and GDP on model training in a toy environment, which allowed us to simply visualize the effect of DP application, we performed a simulation using synthetic datasets. Thus, we generated two synthetic datasets, each representing data from different clients. Then, we employed a neural network model, consisting of two layers, to learn from this data. The model was trained separately on each client’s dataset, with LDP applied by adding noise to the gradients during training. This technique is aimed at protecting individual data points’ privacy by ensuring the model’s updates do not reveal sensitive information. After training the models with LDP, we have aggregated them to form a global model. For the GDP, we have added noise during the aggregation of the weights for the global model update to analyze its impact. The addition of noise is a crucial step in enhancing privacy, as it obscures the contributions of individual clients’ data to the global model.

**Fig. 5:**
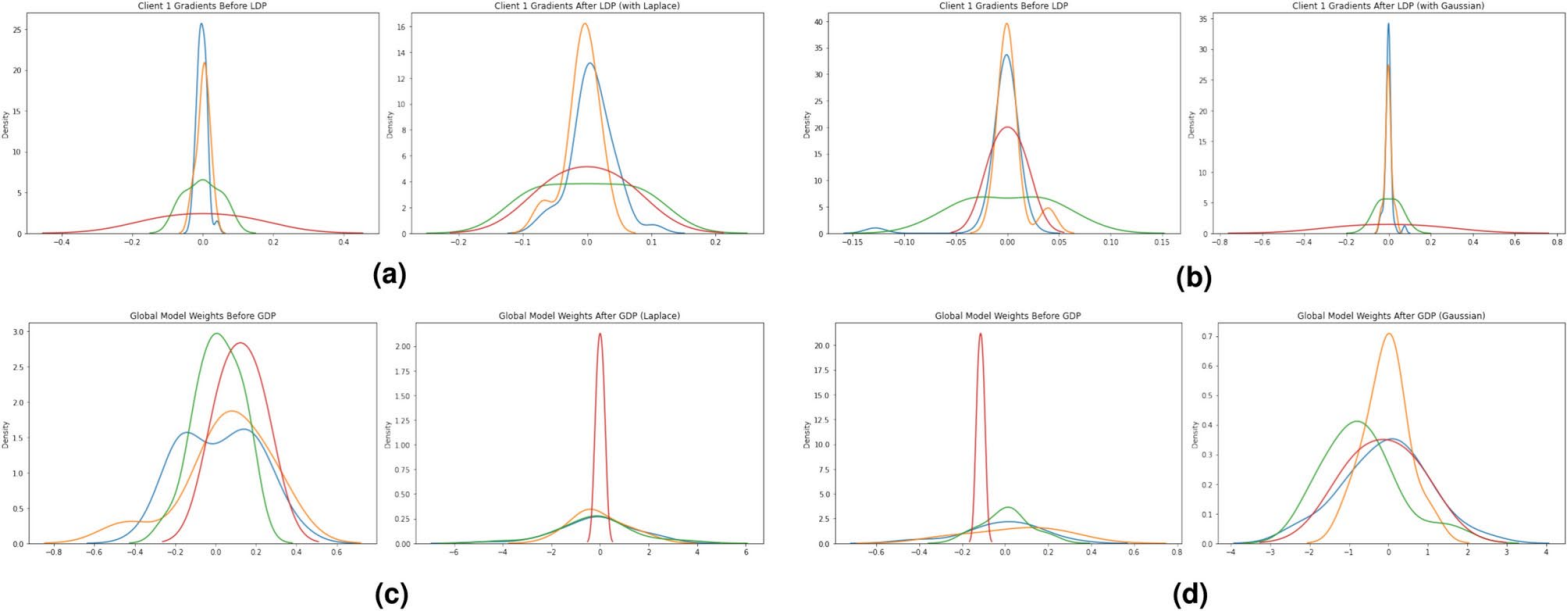
Figures showing the effects of LDP and GDP with Laplace and Gaussian noises.

We have considered two kinds of noises, the Gaussian and the Laplacian ones, for both LDP and GDP. Their formulation is reported next:

3 $$\begin{aligned} \text {Noise} = \text {Laplace}\left( 0, \frac{\Delta f}{\varepsilon }\right) \end{aligned}$$

4 $$\begin{aligned} \text {Noise} = \mathcal {N}\left( 0, \frac{\Delta f}{\varepsilon }\right) \end{aligned}$$

where $\Delta f$ represents the sensitivity (that is a measure of the maximum change in the output of a function due to a change in a single input, used to scale the noise added for achieving differential privacy) of the function and $\varepsilon$ is the privacy budget (that is a privacy parameter quantifying the privacy loss, lower values of $\varepsilon$ indicate stronger privacy guarantees). Figure 5 depicts how gradients change after LDP with Laplacian noise (Fig. 5a), LDP with Gaussian noise (Fig. 5b), GDP with Laplacian noise (Fig. 5c) and GDP with Gaussian noise (Fig. 5d) application, showing that there is a change in the gradients that are sent over the network.

To determine how LDP and GDP impact accuracy, we used the MNIST dataset to train two clients: one with IID data and the other with non-IID data. In our experiment, we used LDP by adding noise to the model parameters of each client before sending them to the server. The noise addiction is performed during model local training on each client’s device. This approach ensured privacy at the local level before any updates were shared with the central server. The server then aggregated these noisy parameters to update the global model. We evaluated the results and observed the accuracy. Next, we trained the same environment using GDP instead of LDP, because we wanted to see the different impact of the techniques. This was accomplished by iterating over the global updates and injecting noise into each parameter, with the noise generated based on the specified sensitivity and $\varepsilon$ values. For all the considered DP techniques analyzed, we have considered both Gaussian and Laplacian noises, whose formulations are described by Equations (3) and (4) respectively.

**Table 7:** Impact of LDP on global model accuracy.

$\varepsilon$	$\Delta f$	Setting	Epoch 1 (%)	Epoch 2 (%)	Epoch 3 (%)	Epoch 4 (%)	Epoch 5 (%)	Average accuracy (%)
		Before LDP	88.84	94.20	95.68	96.44	96.72	96.72
1	1	Gaussian LDP	10.43	10.07	10.32	10.32	10.32	**10.32**
		Laplace LDP	10.92	10.29	9.09	5.76	9.83	9.83
5	1	Gaussian LDP	19.01	17.80	19.85	24.36	15.23	15.23
		Laplace LDP	15.95	22.62	28.09	27.93	29.56	**29.56**
10	1	Gaussian LDP	29.13	44.17	37.19	32.14	47.57	47.57
		Laplace LDP	34.04	29.53	38.08	57.20	72.02	**72.02**
15	1	Gaussian LDP	37.21	21.60	41.28	51.07	47.56	47.56
		Laplace LDP	71.93	54.74	49.99	58.06	76.11	**76.11**

In the “Setting” column, “Before LDP” refers to the framework without the inclusion of any DP technique. The best average accuracy among all the considered settings is underlined, while, the best average accuracy for each parameter configuration is highlighted in bold.

**Table 8:** Impact GDP on global model accuracy

$\varepsilon$	$\Delta f$	Setting	Epoch 1 (%)	Epoch 2 (%)	Epoch 3 (%)	Epoch 4 (%)	Epoch 5 (%)	Average accuracy (%)
		Before GDP	88.84	94.20	95.68	96.44	96.72	96.72
1	1	Gaussian GDP	13.41	9.80	9.80	9.80	9.80	**9.80**
		Laplace GDP	8.75	9.74	10.09	9.82	9.58	9.58
5	1	Gaussian GDP	10.58	17.41	20.54	31.39	31.75	**31.75**
		Laplace GDP	10.0	17.55	15.36	14.73	8.68	8.68
10	1	Gaussian GDP	33.82	35.76	34.24	35.34	44.12	44.12
		Laplace GDP	31.31	46.07	60.33	48.52	58.43	**58.43**
15	1	Gaussian GDP	64.69	73.05	67.32	62.07	55.94	**55.94**
		Laplace GDP	31.08	32.45	40.13	44.36	32.45	32.45

In the “Setting” column, “Before GDP” refers to the framework without the inclusion of any DP technique. The best average accuracy among all the considered settings is underlined, while, the best average accuracy for each parameter configuration is highlighted in bold.

The results of the application of the two considered kinds of noise under different parameters configuration for LDP and GDP are reported respectively in Tables 7 and 8, where, in the “Setting” column, Before LDP (GDP respectively) refers to the environment without the application of DP. We can observe how, as expected, the best accuracy is achieved when we do not apply DP because the weights are not perturbed. Additionally, for both LDP and GDP, an increase in the $\varepsilon$ parameter value meanly corresponds to an increase in the registered accuracy. This behaviour suggests that there is a need for a trade-off between framework privacy level and performance, which deserves attention because it is not easy to have a measure directly quantifying the reached level of privacy. Finally, we can notice how, in this experiment, when we apply LDP, employing Laplace noise seems to obtain better results, while, for GDP, seems to be better to use the Gaussian noise.

### Resources constraints

Resource constraints significantly influence the efficiency of FL environments, particularly when clients with limited computational capabilities, known as “stragglers”, prolong the training process. Network bandwidth limitations and communication losses further complicate timely updates to the global server, which are crucial for synchronizing and aggregating model updates. Although we are not dealing directly with communication protocols or network bandwidth, we have simulated a FL environment with three clients to observe the behavior and importance of computational power.

Our simulation in a PyTorch-based FL environment, involving three clients with varied computational resources, highlighted these challenges. To observe the different amounts of resources available to each local client, we assigned different levels of computational resources (0.5, 1, 1.5) to each model to represent devices with varying capabilities. We simulated the FL environment by loading the MNIST dataset and splitting it into training and validation sets. Each client’s dataset was then prepared from the training set. We assigned different computational powers to each client, affecting their batch sizes and the number of training epochs. Thus, the training duration for each client was recorded to illustrate the impact of computational power.

**Fig. 6:**
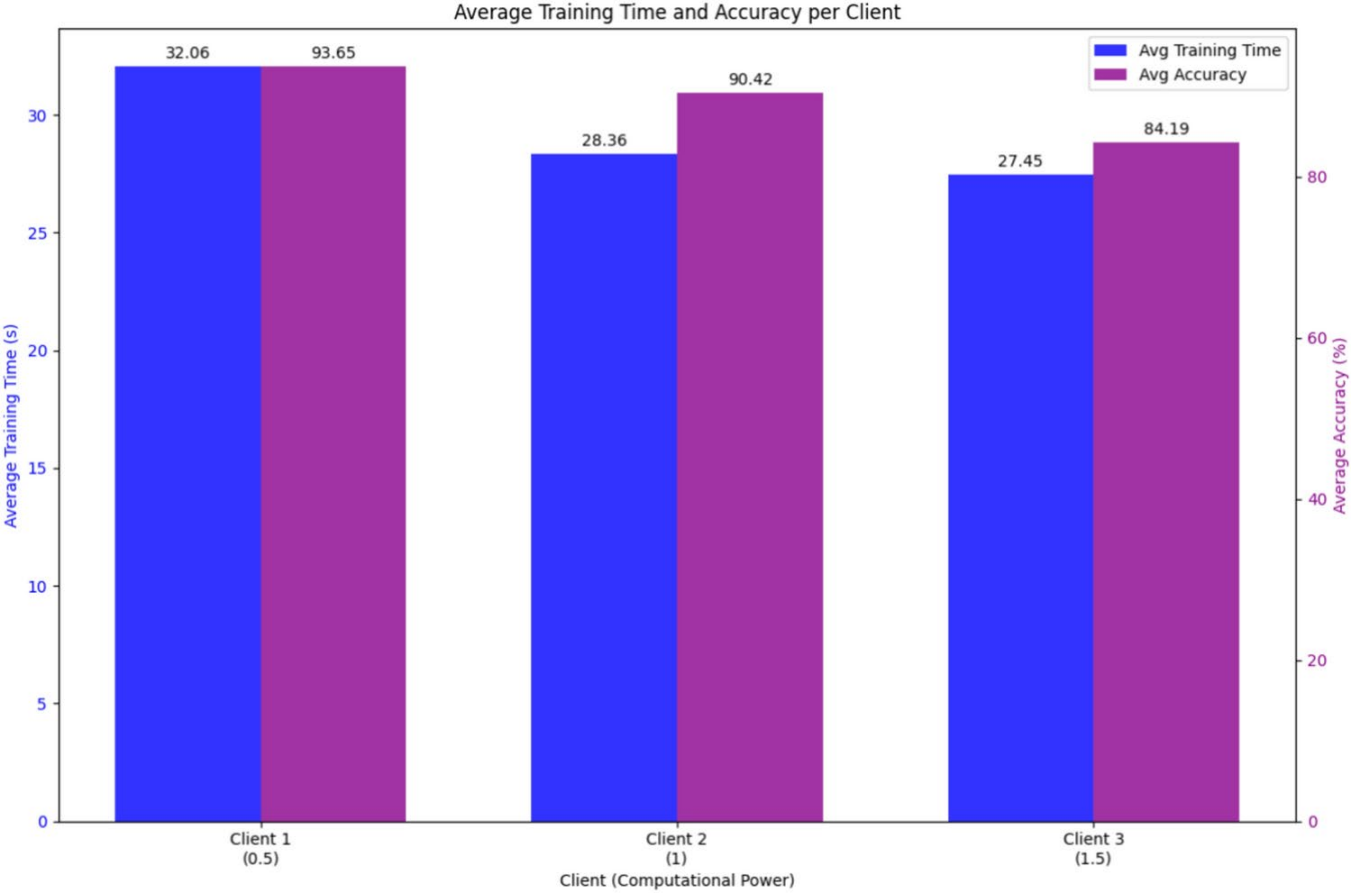
Results of the tests concerning Resource Constraints. The blue bars report the average training time, while the purple ones report the average accuracy.

The results, reported in Fig. 6, show that while all devices achieved similar final accuracy, those with more resources finished training much faster. This suggests that, as expected, computational resources can significantly impact training speed, but they do not necessarily affect the final outcome, and underscores the importance of optimizing computational resources to improve FL system performances.

**Fig. 7:**
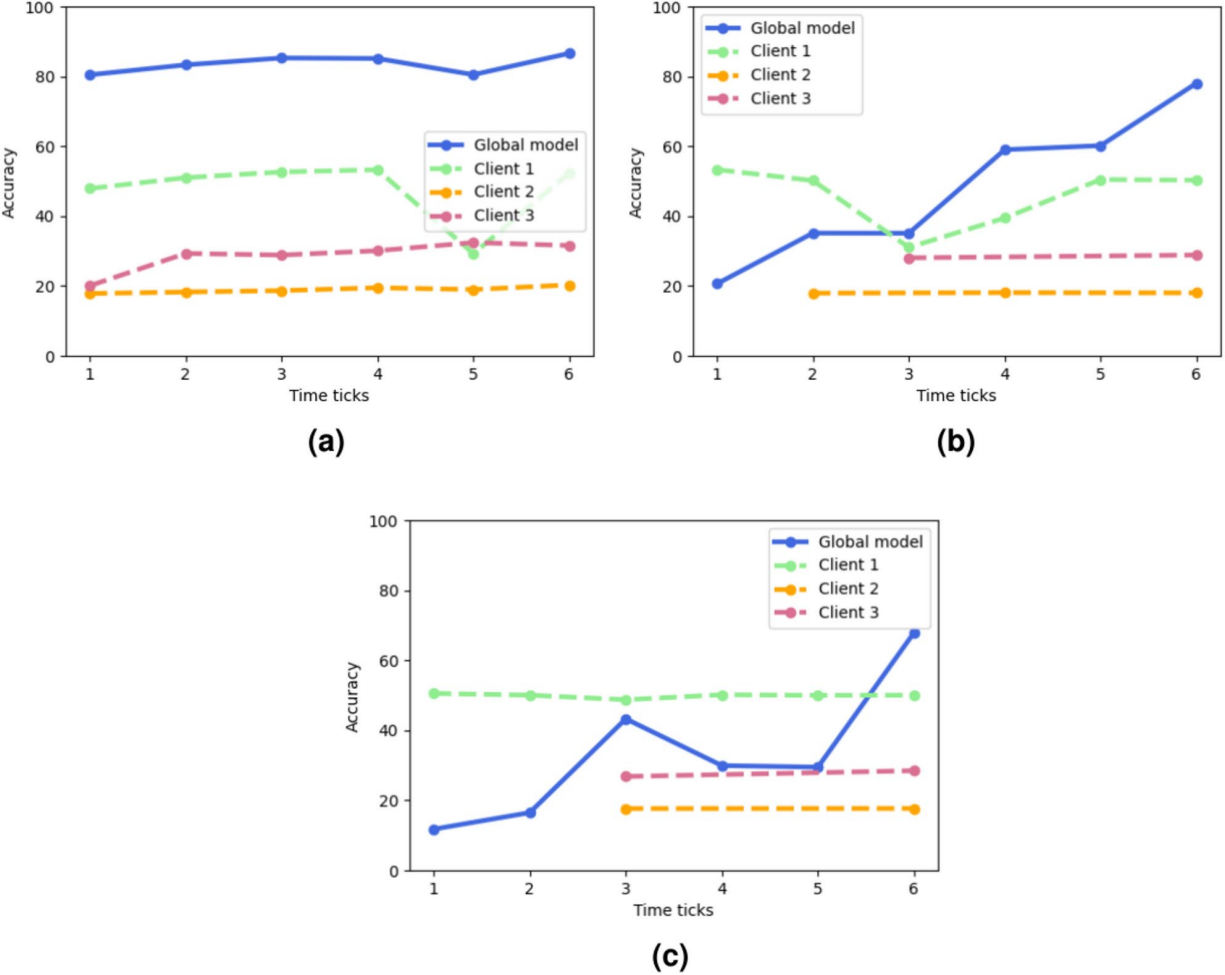
Global model and local models accuracies for three different FL framework settings. (**a**) Shows the results for an uniform setting involving three high-performing clients, (**b**) refers to a setting involving an high-performing client, a middle-performing client and a low-performing client, finally, (**c**) refer to a setting involving one high-performing client and two low-performing clients.

Thus, in order to analyze in more detail the effect that local clients with different computing capabilities have in a FL environment, we performed a test involving three settings. We considered an environment in which the updated weights can be sent by the models at specific time ticks and, under this assumption, we characterized local clients with different computing capabilities. In particular, high-performing clients send updated weights at each time tick, middle-performing clients send updates every two time ticks and low-performing clients send updates every three time ticks. We analyzed three different settings: the first one, whose results are reported in Fig. 7a, considers a FL framework composed of three high-performing local clients, the second one, whose results are depicted by Fig. 7b, considers a FL framework having one high-performing local client, one middle-performing local client and one low-performing local client, finally, the last setting, whose results are depicted by Fig. 7c, considers a FL framework characterized by one high-performing and two low-performing local clients. The plots reported in Fig. 7, in which each weight exchange between one local model and the global model is highlighted by a dot, show how having devices with different computing capabilities affects the performances of the global model. Indeed, despite not showing significative decreases in the final accuracy, the global model accuracy has a slower increasing trend in the second setting (Fig. 7b) or shows a less stable trend (Fig. 7c) when there is a huge gap in computing capabilities. In the time steps in which the global model does not receive updates by the stragglers model, it tends to overfit over the high-performing clients.

## Discussion

The practical analysis of FL performed in the paper offers some insights into understanding some relevant issues in FL. By enhancing data privacy and security, FL becomes more viable for sensitive applications, such as healthcare and finance, where protecting user data is essential. These strategies enable the development of more inclusive and representative ML models, capable of learning from a broad spectrum of real-world data. Our empirical analysis of data heterogeneity and model complexity highlights the critical role of data in developing globally representative ML systems. Thus, one of the big open challenges is to promote updates based on “good data” while trying to mitigate the influence of updates based on data characterized by problems related either to distribution or quality.

Our simulation highlights that computational resources significantly impact training speed in FL, even though they do not necessarily affect the final outcome much. As FL continues to evolve, future research might focus on innovative solutions that further reduce communication overhead and computational demands, making FL accessible to a wider range of devices and networks, since this would make ML more largely adaptable, allowing even devices with limited resources to contribute to and benefit from shared learning.

While it is becoming more and more largely accepted the viability of FL as a transformative approach to ML, capable of addressing some of the most pressing challenges in data privacy, security, and data heterogeneity, our simple experimental campaign has also shown that there is still a lot to do to improve FL technology, which, potentially, is the key to a paradigm shift in Deep Learning.

In discussing the broader implications of our findings, it has become clear that addressing the challenges of FL requires not only theoretical insights but also development of the practical tools and environments. While our research did not directly employ these technologies, our analysis pointed to several leading tools and libraries that hold promise for advancing FL projects. TensorFlow Federated (TFF) and PySyft, for example, offer robust frameworks designed with privacy and security at the forefront, making them suitable for projects that prioritize these concerns. TFF’s ability to deploy models on real-world datasets^34^ and PySyft’s focus on encrypted, privacy-preserving computations^35^ suggest their potentialities in overcoming some of the challenges we have identified.

Furthermore, tools like FATE^4^ and PaddleFL^36^ cater to the specific needs of industries such as finance and healthcare, where data privacy is paramount. Their development underscores the industry’s move towards more secure and compliant FL solutions. The framework-agnostic library Flower^37^ and the benchmarking framework LEAF^38 ^also emerge as valuable resources. Flower’s flexibility in FL research and LEAF’s provision of realistic evaluation benchmarks align with the need for adaptable and rigorous FL methodologies.

These tools and libraries represent the cutting edge of FL technology, capable of addressing the decentralized data training challenges also highlighted in our analyses. As we look to the future, leveraging these resources will be crucial for researchers and practitioners aiming to navigate the complexities of FL, enhancing the efficiency, scalability, and security of FL systems.

## Conclusion

By exploring the rapidly developing field of FL, this paper provides some add-ons to our understanding of the documented issues that arise in the practical development of FL models by mustering them in a single work. Following this, we analyzed some potential solutions to address these challenges. We effectively experimented with the challenges of data heterogeneity, model aggregation, data privacy, and diversified local clients computing capabilities that hamper FL’s integration into real-world applications. Through experimentation and analysis of existing studies, we observed the impact that some of the strategies available in literature in the enhancement of FL’s performance, usability, and security, which can impact the future of ML development.

Our investigation into client weighting and model personalization strategies underscored their critical role in mitigating data heterogeneity challenges. By practically experimenting with them in simple environments we designed, we brought out their impact on model performance across diverse data distributions. Also, we remarked on how effective aggregation techniques are crucial for achieving global model accuracy. These techniques are essential for handling data in different forms and quantities from various clients in FL frameworks.

The importance in FL of protecting data privacy effectively led us to investigate how differential privacy could be applied in this specific case. Our studies confirm that both local and global differential privacy methods demonstrably enhance data security. This approach prioritizes user privacy, allowing researchers to train models while adhering to strong privacy guarantees. This balancing act underscores the importance of privacy-preserving FL in sensitive domains, offering a promising approach to privacy.

To summarize, this study sheds some light on practical considerations for FL by examining its behaviour in diverse scenarios. Drawing upon diverse data partitions and an understanding of existing FL challenges, our research offers some insights into the considered realm. We demonstrated how existing techniques, when effectively applied, promise to enhance FL performance in practical settings. This facilitates the way for broader adoption and increased effectiveness of decentralized machine learning intelligence.

Our future work will delve into exploring novel encryption techniques and aggregation methods, aiming to enhance the privacy and efficiency of FL systems. Additionally, we will investigate the application of FL in emerging fields, such as edge computing and the Internet of Things, to fully realize its potential in driving innovation and advancing technology for societal benefit.

## Data Availability

The data that support the findings of this study are openly available. The MNIST dataset is available in the LEAF benchmark at https://leaf.cmu.edu/. The CIFAR-10 dataset is available at https://www.cs.toronto.edu/~kriz/cifar.html.

## References

[1] Alaqra, A. S., Kane, B. & Fischer-Hübner, S. Machine learning-based analysis of encrypted medical data in the cloud: Qualitative study of expert stakeholders’ perspectives. *JMIR Hum. Fact.* **8**, e21810 (2021). 10.2196/21810 PMC8485196 34528892

[2] Brauneck, A. et al. Federated machine learning, privacy-enhancing technologies, and data protection laws in medical research: Scoping review. *J. Med. Internet Res.* **25**, e41588 (2023). 36995759 10.2196/41588 PMC10131784

[3] Li, L., Fan, Y. & Lin, K.-Y. A survey on federated learning. in *2020 IEEE 16th International Conference on Control & Automation (ICCA)*, 791–796, 10.1109/ICCA51439.2020.9264412 (2020).

[4] Yang, Q., Liu, Y., Chen, T. & Tong, Y. Federated machine learning: Concept and applications. *ACM Trans. Intell. Syst. Technol. (TIST)*[SPACE]10.1145/3298981 (2019).

[5] Li, T., Sahu, A. K., Talwalkar, A. & Smith, V. Federated learning: Challenges, methods, and future directions. *IEEE Sign. Process. Mag.* **37**, 50–60 (2020).

[6] Bonawitz, K. *et al*. *Towards federated learning at scale: System design.* **1**, 374–388 (2019).

[7] Wang, S. et al. Adaptive federated learning in resource constrained edge computing systems. *IEEE J. Sel. Areas Commun.* **37**, 1205–1221 (2019).

[8] Smith, V., Chiang, C.-K., Sanjabi, M. & Talwalkar, A. Federated learning: Strategies for improving communication efficiency. arXiv preprint arXiv:1610.05492 (2017).

[9] McMahan, H. B., Moore, E., Ramage, D., Hampson, S. & y Arcas, B. A. Communication-efficient learning of deep networks from decentralized data. in *AISTATS* (2017).

[10] Sweeney, L. k-anonymity: A model for protecting privacy. *Internat. J. Uncertain. Fuzziness Knowl.-Based Syst.* **10**, 557–570. 10.1142/S0218488502001648 (2002).

[11] Dwork, C. Differential privacy: A survey of results. *Internat. J. Uncertain. Fuzziness Knowl.-Based Syst.* **10**, 557–570. 10.1142/S0218488502001648 (2008).

[12] Konečný, J., McMahan, H. B., Ramage, D. & Richtárik, P. Federated learning: Strategies for improving communication efficiency. in *NIPS Workshop on Private Multi-Party Machine Learning* (2016).

[13] Guo, S. et al. Sandbox computing: A data privacy trusted sharing paradigm via blockchain and federated learning. *IEEE Trans. Comput.* **72**, 800–810 (2022).

[14] Song, J., Wang, W., Gadekallu, T. R., Cao, J. & Liu, Y. Eppda: An efficient privacy-preserving data aggregation federated learning scheme. *IEEE Trans. Netw. Sci. Eng.* **10**, 3047–3057. 10.1109/TNSE.2022.3153519 (2023).

[15] Jia, B. et al. Blockchain-enabled federated learning data protection aggregation scheme with differential privacy and homomorphic encryption in iiot. *IEEE Trans. Industr. Inf.* **18**, 4049–4058. 10.1109/TII.2021.3085960 (2022).

[16] Pillutla, K. *et al.* Federated learning with partial model personalization. In Chaudhuri, K. *et al.* (eds.) *Proceedings of the 39th International Conference on Machine Learning*, vol. 162 of *Proceedings of Machine Learning Research*, 17716–17758 (PMLR, 2022).

[17] Xiong, Z., Cai, Z., Takabi, D. & Li, W. Privacy threat and defense for federated learning with non-i.i.d. data in AIoT. *IEEE Trans. Ind. Inform.* **18**, 1310–1321. 10.1109/TII.2021.3073925 (2022).

[18] Li, Z., Lin, T., Shang, X. & Wu, C. Revisiting weighted aggregation in federated learning with neural networks (2023). arXiv:2302.10911.

[19] Zhao, Y. *et al.* Federated learning with non-iid data, 10.48550/ARXIV.1806.00582 (2018).

[20] Mothukuri, V. et al. A survey on security and privacy of federated learning. *Futur. Gener. Comput. Syst.* **115**, 619–640. 10.1016/j.future.2020.10.007 (2021).

[21] Li, Q. et al. A survey on federated learning systems: Vision, hype and reality for data privacy and protection. *IEEE Trans. Knowl. Data Eng.* **35**, 3347–3366. 10.1109/TKDE.2021.3124599 (2019).

[22] Kairouz, P. et al. Advances and open problems in federated learning. *Found. Trends Mach. Learn.* **14**, 1–210 (2021).

[23] Duan, Q. et al. Combining federated learning and edge computing toward ubiquitous intelligence in 6g network: Challenges, recent advances, and future directions. *IEEE Commun. Surv. Tutor.* **25**, 2892–2950. 10.1109/COMST.2023.3316615 (2023).

[24] LeCun, Y., Cortes, C., Burges, C. *et al.* Mnist handwritten digit database (2010).

[25] Caldas, S. *et al.* Leaf: A benchmark for federated settings (2019). arXiv:1812.01097.

[26] Krizhevsky, A. et al. *Learning Multiple Layers of Features from Tiny Images. Tech. Rep.* (University of Toronto, 2009).

[27] Nickparvar, M. Brain tumor MRI dataset. https://www.kaggle.com/datasets/masoudnickparvar/brain-tumor-mri-dataset/data (2023). Accessed 19 Sept 2024.

[28] Zhang, F. et al. Recent methodological advances in federated learning for healthcare. *Patterns* **5**, 101006 (2024). 39005485 10.1016/j.patter.2024.101006 PMC11240178

[29] Mendieta, M. *et al.* Local learning matters: Rethinking data heterogeneity in federated learning. CoRR. abs/2111.14213 (2021). arXiv:2111.14213.

[30] Ye, M., Fang, X., Du, B., Yuen, P. C. & Tao, D. Heterogeneous federated learning: State-of-the-art and research challenges. *ACM Comput. Surv.* **56**, 1–44. 10.1145/3625558 (2023).

[31] Lee, S., Yu, M., Yoon, D. & Oh, S. Can hierarchical client clustering mitigate the data heterogeneity effect in federated learning? in *2023 IEEE International Parallel and Distributed Processing Symposium Workshops (IPDPSW)*, 799–808. 10.1109/IPDPSW59300.2023.00134 (2023).

[32] Wilbik, A., Pekala, B., Dyczkowski, K. & Szkoła, J. A comparison of client weighting schemes in federated learning. in Atanassov, K. T. *et al.* (eds.) *Uncertainty and Imprecision in Decision Making and Decision Support: New Advances, Challenges, and Perspectives*, 116–128 (Springer Nature Switzerland, Cham, 2023).

[33] Wei, K. *et al.* Federated learning with differential privacy: Algorithms and performance analysis (2019). arXiv:1911.00222.

[34] Abadi, M. *et al.* Tensorflow: Large-scale machine learning on heterogeneous distributed systems. arXiv preprint arXiv:1603.04467 (2016).

[35] Ryffel, T. *et al.* Pysyft: A python library for secure and private deep learning. arXiv preprint arXiv:1811.04017 (2018).

[36] Ma, L. *et al.* Paddlefl: An industrial grade federated learning framework. arXiv preprint arXiv:2007.01825 (2020).

[37] Beutel, D. J. *et al.* Flower: A friendly federated learning research framework. arXiv preprint arXiv:2007.14390 (2020).

[38] Caldas, S. *et al.* Leaf: A benchmark for federated settings. arXiv preprint arXiv:1812.01097 (2018).

